# Micro-Analytical Lab-on-a-Tip: Advances and Perspectives

**DOI:** 10.1021/acs.analchem.5c04914

**Published:** 2025-09-30

**Authors:** Panagiota M. Kalligosfyri, Shohreh Madani, Antonella Miglione, Wanda Cimmino, Stefano Cinti, Amir Hatamie

**Affiliations:** 1 Department of Pharmacy, 9307 University of Naples Federico II, Naples 80131, Italy; 2 Department of Chemistry, Institute for Advanced Studies in Basic Sciences (IASBS), Zanjan 45137-66731, Iran; 3 Bioelectronics Task Force at University of Naples Federico II, Naples 80126, Italy; 4 Sbarro Institute for Cancer Research and Molecular Medicine, Center for Biotechnology, College of Science and Technology, Temple University, Philadelphia, Pennsylvania 19122, United States

## Abstract

The rapid advancement of miniaturized analytical tools has revolutionized modern chemical analysis, with pipet tip-based sensors emerging as versatile, low-cost, and portable platforms for on-site detection. By repurposing standard plastic pipet tips as microreactors or sensor holders, these devices integrate electrochemical and optical elements to support applications in biomedical diagnostics, environmental monitoring, and food safety. Recent innovations include the incorporation of microelectrodes, nanomaterials, and 3D-printed components, enabling highly sensitive electroanalysis with minimal sample and reagent consumption. Optical systems, such as colorimetric and fluorescence-based assays, further enhance functionality by allowing rapid, visually interpretable, and multiplexed detection of ions, biomolecules, nucleic acids, and synthetic additives. Approaches based on filter paper, hydrogels, polymer networks, and additive manufacturing techniques have expanded the functional versatility of these devices by supporting sample filtration, reagent storage, and controlled reactions. Smart materials and magnetic separation further improve selectivity and sensitivity, while integration with smartphones enables real-time, decentralized diagnostics. These lab-on-a-tip systems align with green analytical chemistry principles by reducing chemical waste and promoting sustainable, user-friendly practices. Their affordability, ease of use, and adaptability make them especially valuable in resource-limited settings. This perspective presents a comprehensive overview of recent developments in pipet tip-based analytical devices, highlighting their transformation from simple laboratory tools into multifunctional sensing platforms. By summarizing key trends, design strategies, and practical applications, it underscores their pivotal role in shaping next-generation, environmentally friendly, and accessible point-of-care technologies.

## Graphical abstract

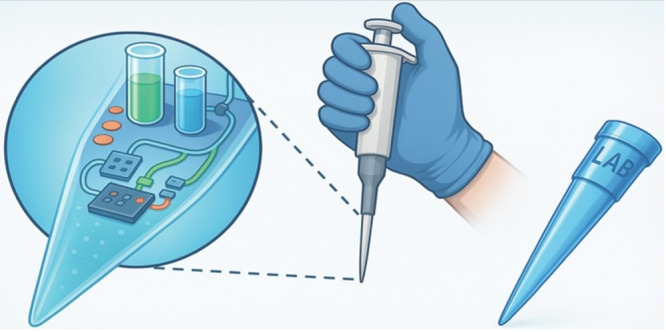

## Introduction

In modern analytical chemistry, the design and fabrication of miniaturized, cost-effective sensing tools that focus on affordability, applicability, and multifunctionality have gained significant attention across various fields, including biomedical, environmental, security, and food analysis.^1–4^ The growing demand for these tools is largely driven by their portability, which enables chemical analysis to transition from laboratory settings to real-world and on-site applications. Examples of such tools include strip tests, pregnancy test strips, portable glucose sensors, and commercial hand-held pH meters.^4–8^ The interest in these miniaturized tools stems from their numerous advantages, including broad applicability and alignment with green chemistry principles.

One of the primary benefits of miniaturized analytical tools is their ability to perform electrochemical and optical measurements using extremely small sample volumes. This is particularly valuable for analyzing low-volume target samples, such as scarce biological fluids like human tears and saliva, which contain a variety of biomarkers important for diagnostic purposes. These tools are also well-suited for analyzing microscale environmental, industrial, and agricultural samples, including rain, aerosols, and wastewater droplets, as well as in forensic investigations and the detection of illicit drug samples.^9,10^ Additionally, in biological analyses such as neuroscience research, these tools enable in situ analysis of tiny biosamples, including single cells,^11,12^ which is crucial for disease research and drug development.

From a green chemistry perspective, miniaturized tools often require minimal reagent volumes, or, in some cases, are entirely reagent-free, significantly reducing chemical consumption and hazardous waste generation.^13^ As such, they align well with the principles of green analytical chemistry, offering more sustainable alternatives to conventional analytical tools while also reducing the overall cost of analysis.

Another major advantage of miniaturized systems is their capability for real-time, on-site, and localized analysis, which saves both time and costs. These tools often eliminate or reduce the need for extensive sample preparation and transportation to well-equipped laboratories, making rapid and efficient analysis possible while conserving resources. Moreover, most of these tools are user-friendly and easy to operate, allowing even untrained individuals to use them. This is particularly important in low-resource settings or regions lacking access to advanced and expensive laboratory facilities, making them highly beneficial for underdeveloped areas and remote locations.^14,15^ In advanced healthcare systems, remote diagnostic tools play a key role and can significantly improve people’s quality of life.

Finally, cost-effectiveness is a key factor driving the adoption of these portable sensors. Beyond their low reagent consumption and simplified sample preparation, many of these tools have low fabrication costs, have minimal maintenance requirements, and are often designed for single-use applications. Despite their simplicity, they often offer acceptable—if not high—sensitivity and accuracy, comparable to advanced analytical instruments, which are typically expensive, require specialized facilities, and demand trained professionals for operation.^16,17^

In recent decades, significant advancements in micro- and nanotechnology have greatly enhanced the performance of miniaturized analytical tools across various domains, including electronics, machinery, and sensing.^18^ Innovations in nanomaterials have led to improvements in sensor design, fabrication techniques, and surface modifications, all contributing to increased sensitivity and selectivity, ultimately resulting in more efficient miniaturized sensors. Additionally, progress in nano- and microfabrication has enabled the development of a wide variety of small, portable microfluidic sensing devices.^19^

Additionally, new fabrication technologies and novel materials, including plastics and polymers like polydimethylsiloxane (PDMS) and polytetrafluoroethylene (PTFE), have expanded the possibilities for developing new sensor substrates and templates.^20^ One particularly interesting material is paper, which has been explored as a substrate to replace traditional microfluidic materials such as glass, silicon, quartz, and even polymers. Compared to these conventional materials, paper is natural, abundant, low-cost, biodegradable, biocompatible, and environmentally friendly.^21,22^ A unique feature of paper in sensor design and fabrication is its ability to transport liquid samples through capillary action within the paper matrix, without the need for any external force, a key factor in lateral flow tests and similar applications. Furthermore, it can be easily cut into various formats and shapes, making it highly suitable for applications in purification, separation, microfluidic sensors, and green analytical chemistry. All of these factors make paper an excellent choice for the fabrication of low-cost, miniaturized analytical sensors.^23,24^

In parallel with advances in miniaturized sensor design, recent developments in conductive inks and modern printing technologies, such as 3D printing, have enabled researchers to fabricate sensors and thin-film electrodes in various shapes and sizes on a wide range of substrates, including paper, plastics, textiles, and even robotic structures.^3,25,26^

As one of the latest innovations in this field, miniaturized analytical tools have been integrated with various platforms and automated or controlled by artificial intelligence (AI), resulting in the development of highly advanced optical and electrochemical sensing systems. This integration holds notable potential to revolutionize sensing technologies by enabling autonomous operation, data processing, and remote sensing capabilities.^27,28^

In this perspective, we focus on a particularly interesting, simple, and low-cost category of miniaturized sensors fabricated using common plastic pipet tips. These pipet tips, widely used in both manual and automated microsampling systems, have recently gained attention as versatile platforms for sensor development. Over the past decade, researchers have explored their potential beyond traditional sample handling, utilizing them as microtemplates for the fabrication of a variety of optical and electrochemical sensors. It highlights recent trends, technological advancements, and diverse applications of plastic pipet tips in analytical chemistry. We demonstrate how these inexpensive and readily available tools can be transformed into portable optical and electrochemical sensors, showcasing their potential to revolutionize modern analytical techniques.

## Sensing Strategies Using Micropipette Tip Technologies

### Electroanalysis with Lab-on-a-Tip

To meet the diverse requirements for designing and fabricating pipet-based tips tailored to specific applications, various plastic tip sizes, configurations, and quantities have been utilized. In this context, lab-on-a-tip systems are innovatively designed, modified, and applied for sensing, serving as sensor holders, substrates, and more. This section reviews commonly reported structures, their modifications and integrations with other analytical systems, and different models and components of analytical pipet-based sensors, excluding sampling.

The fundamental component of this category of analytical tools is the micropipette tip—a common laboratory tool available in large quantities and at affordable prices in various sizes and volumes. These tips are primarily made from polypropylene, a highly stable and chemically inert material. Depending on their size, micropipette tips can accommodate sample volumes ranging from the microliter to milliliter scale and typically feature an orifice in the millimeter range. However, smaller tips are generally preferred for fabricating microelectrodes and sensors.^29,30^

Three primary approaches are commonly used to fabricate electrodes. The first and second approaches use the pipet tip as a holder, either filled with conductive material or securing inserted electrodes, while the third approach integrates the electrodes within the tip, forming a self-contained electrochemical cell. In the first approach, the pipet tip is entirely filled with a conductive material, with the orifice size controlling the electrode dimensions. For example, Dejmkova et al.^31^ utilized 100 μL plastic pipet tips filled with carbon paste electrodes to determine chlortoluron in environmental samples (Figure 1A). In this setup, the tip served solely as a holder, while the orifice defined the physical surface of the electrode. Similarly, to enhance selectivity, Zhang et al.^32^ developed a carbon nanotube-based paste containing a molecularly imprinted polymer (MIP) to fill the pipet tip (Figure 1B), enabling highly selective electroanalysis of cannabis components such as tetrahydrocannabinol (THC).

**Figure 1:**
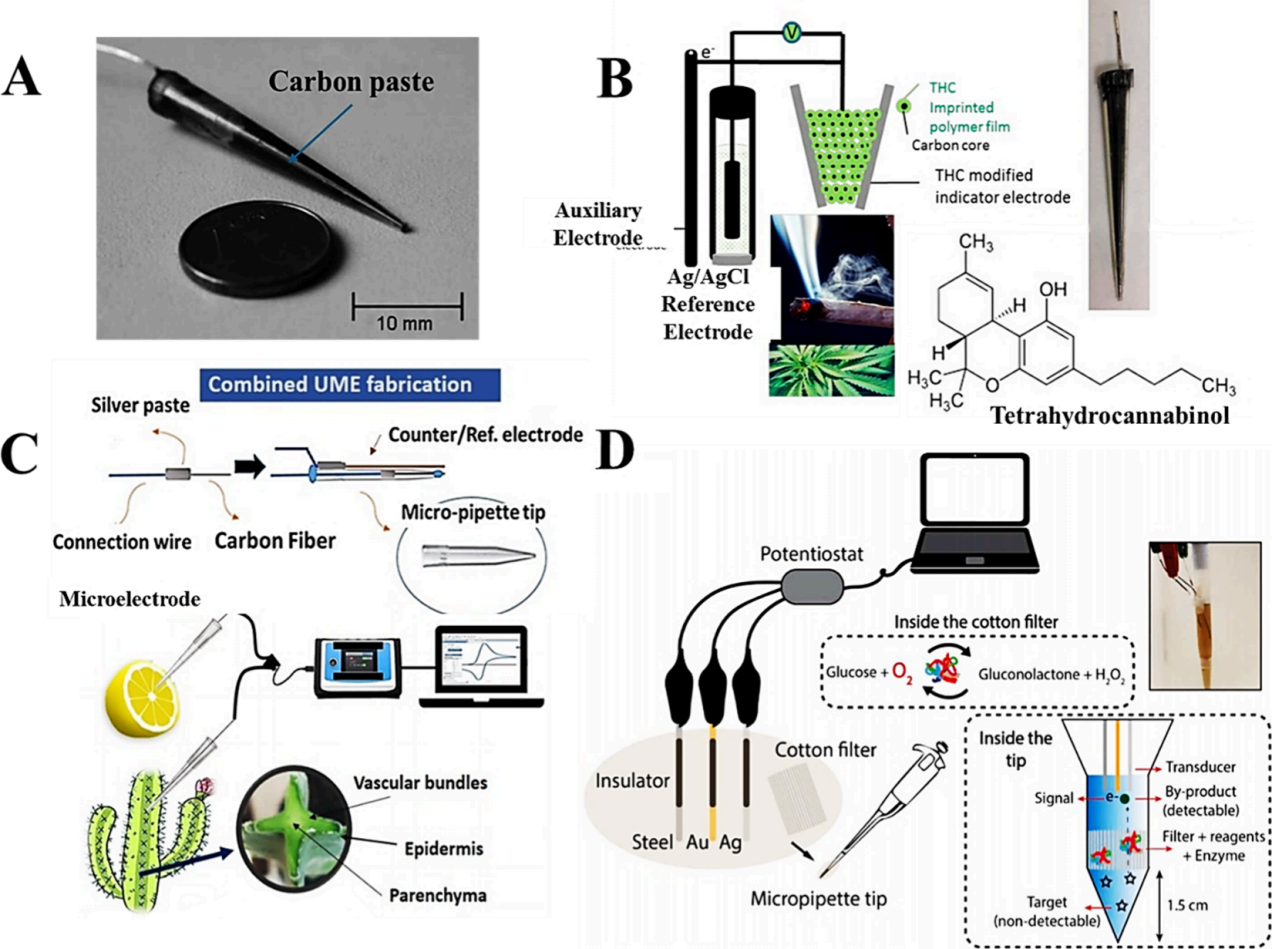
(A) Miniaturized micropipette tip electrode filled with carbon paste for electrochemical sensing. Reproduced from Dejmkova et al. 2013 (ref 31). Copyright 2013 published by Wiley. (B) Micropipette-based sensors filled with carbon nanotubes (CNTs) or carbon beads, combined with a molecularly imprinted polymer (MIP) selective for tetrahydrocannabinol (THC), an illicit drug. Reproduced from Zhang et al. 2019 (ref 32). Copyright 2019 published by Elsevier (C) Schematic illustration of the fabrication of a combined microelectrode (two-electrode system) (top), along with a schematic representation of its application for in vivo and in vitro analysis of ascorbic acid in lemon and cactus samples. Reproduced from Fathi and Hatamie 2025. (ref 34). Copyright 2025 published by Wiley. (D) Schematic representation of a biolab-on-a-tip system (three-electrode configuration) designed for sampling and glucose biodetection. Reproduced from Cinti et al. 2020 (ref 35). Copyright 2020 published by Elsevier.

In the second approach, the pipet tip functions as a holder for metallic or nonmetallic electrodes. In this configuration, metallic wires are inserted into the tip, and the remaining internal volume is filled with an insulating material, such as epoxy, to secure the wires. For instance, Zhang^33^ inserted a carbon fiber electrode (diameter: 7 μm) into a pipet tip. The carbon fiber electrode (CFE) was connected to a copper wire using silver paint as a conductive link to the potentiostat, while the interior space of the pipet tip was filled with epoxy to secure the electrode in place. This micropipette-tip-based electrochemical device was applied for glucose analysis in plasma. In this setup, only the working electrode was housed inside the tip, whereas other electrodes, such as the reference electrode, were used separately. Using a similar strategy, Fathi and Hatamie^34^ fabricated a portable, two-electrode microsensor for direct sensing of ascorbic acid (vitamin C) in biological systems, such as fruit tissue (e.g., lemon) and plant structures (e.g., cactus arms and trunk). This microsensor analyzes both the concentration and distribution of ascorbic acid within these samples directly. As shown in Figure 1C, a microscale carbon fiber electrode (33 μm in diameter) is coated with a gold nanofilm and connected to a silver wire, which serves as the electrical contact. This electrode is inserted into a micropipette tip, acting as the working electrode holder. Additionally, a silver wire is placed outside the pipet tip to function as a semireference electrode. This two-electrode setup not only serves as an implantable microsensor for ascorbic acid but also reveals significant findings: Ascorbic acid levels are heterogeneously distributed within lemon tissue, a detail that is challenging to determine using other techniques that require sampling and preparation steps. Furthermore, in applications relevant to food quality and the agricultural industry, the fabricated microsensor showed that stored ascorbic acid levels correlate with the color of the lemon, as yellow lemons contain higher levels of ascorbic acid than green ones. It also highlighted how various storage conditions, such as exposing lemons to sunlight or storing them in clear bottles, can affect the ascorbic acid levels in these products. For more applied findings, refer to the original paper. Overall, this approach clearly broadens the application of pipet-based electrochemical sensors that is not possible with a common electrochemical system.

In the third configuration, electrodes are placed within or on the body of the pipet tip and securely fixed. This model features a two- or three-electrode setup, where all electrodes are integrated into a single tip. A key advantage of this design is that the pipet tip remains empty, allowing it to function as both a sample collector and an electrochemical sensor. This enables high-precision microvolume sampling and simultaneous electroanalysis, making it particularly useful for on-site analyses. To date, various two- and three-electrode configurations have been fabricated and applied.

In one of the earliest designs, Cinti et al.^35^ developed a three-electrode configuration in which a gold wire (0.03 mm in diameter) served as the working electrode, a silver wire (0.25 mm in diameter) as the reference electrode, and a steel wire (0.3 mm in diameter) as the counter electrode (Figure 1D). This miniaturized electrochemical tool was applied for amperometric glucose sensing in clinical diagnostics and monitoring. To enhance sensitivity and selectivity, the researchers used an enzyme (glucose oxidase) and an electrode modifier. Unlike conventional biosensors where the enzyme is immobilized directly on the working electrode, this design placed the enzyme inside the pipet tip. A porous filter within the commercial pipet tip served as a substrate for enzyme immobilization. This biofilter, which is commonly available in some commercial pipet tips, facilitated the conversion of glucose in the sample to hydrogen peroxide (H_2_O_2_) as it passed through the filter. The generated H_2_O_2_ was subsequently detected by the gold microelectrode. In another study, Cinti et al.^36^ modified the cotton wool filter inside the pipet tip with an electroactive reagent that was released upon contact with the target analyte (Figure 2A). This tiny filter played a dual role: First, it purified the sample by removing suspended impurities, and second, it acted as a reservoir, releasing preloaded electroactive reagents upon detecting the target analyte.

**Figure 2:**
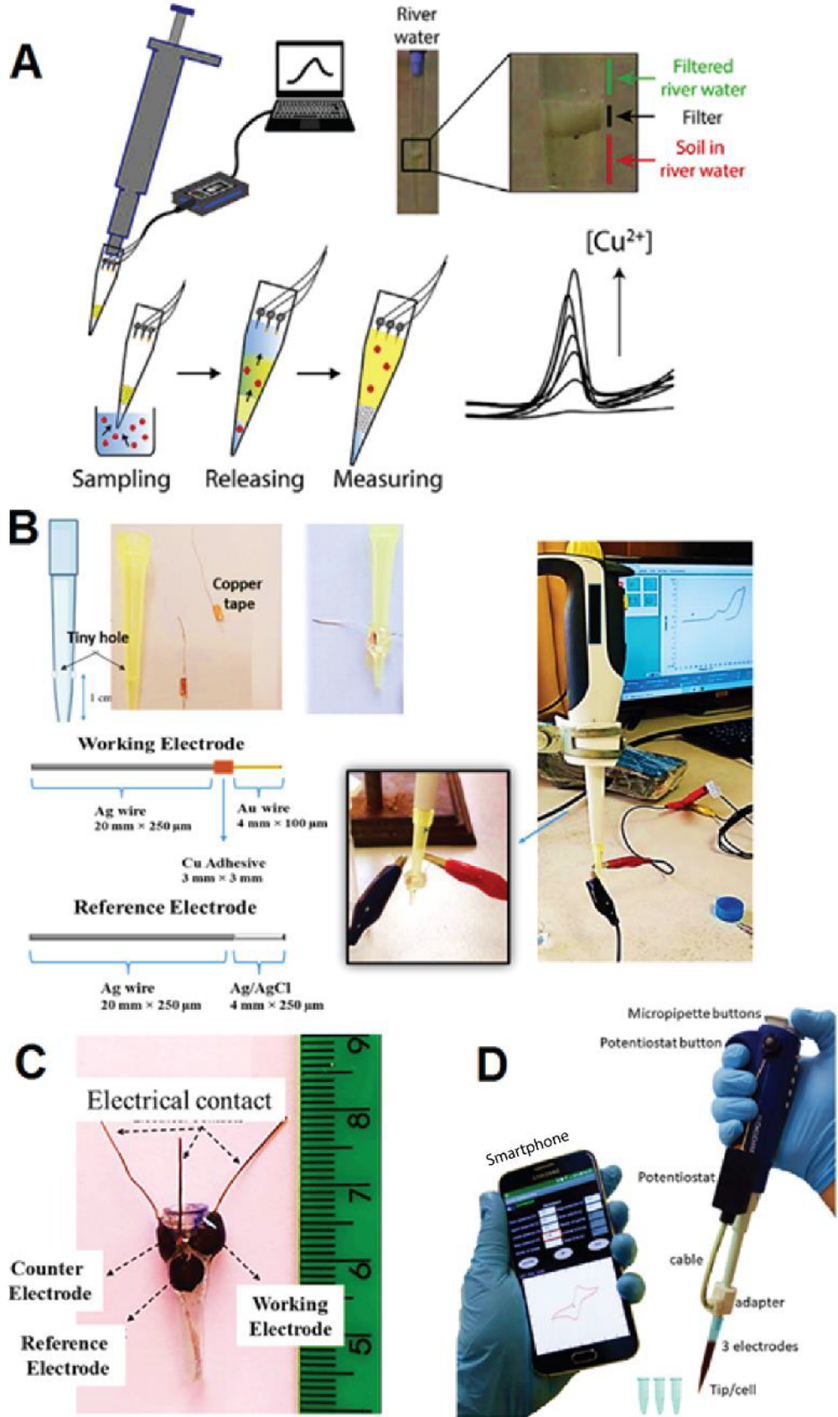
(A) Lab-on-a-microtip system (three-electrode configuration) developed for the electroanalysis of copper ions in river water. Reproduced from Cinti et al. 2018 (ref 36). Copyright 2018 published by Elsevier. (B) Image of the drilled pipet tip (with two holes) for installing two metallic wire electrodes (top left), schematic of the fabricated electrode structure (bottom left), miniaturized electrochemical cell connected to a pipet injector and potentiostat with CV displayed on screen (top right), and electrochemical microscopic imaging of the working electrode surface modified with gold nanoparticles (bottom right). Reproduced from Madani and Hatamie 2024 (ref 37). Copyright 2024 published by the American Society Chemistry. (C) Miniaturized three-electrode electrochemical cell, where all electrodes were fabricated using a 3D pen and printed directly within the walls of a drilled micropipette tip. Reproduced from de Oliveira et al. 2022 (ref 43). Copyright 2022 published by Elsevier. (D) Image of the integrated micropipette–potentiostat–smartphone system, a hyphenated instrument designed for automatic sampling and sensing. Reproduced from Barragan and Kubota 2020 (ref 44). Copyright 2020 published by Elsevier.

More recently, Madani and Hatamie^37^ fabricated a miniaturized electrochemical cell within a single micropipette tip (100 μL capacity) using a two-electrode configuration (working vs semireference electrode). To construct the working electrode, a micro gold wire (100 μm in diameter) was inserted into a predrilled section of the pipet tip and secured with epoxy (Figure 2B). A silver wire (0.25 mm in diameter) was inserted from the opposite side at a predetermined position. To further enhance sensitivity, the working electrode surface was modified with gold nanoparticles (AuNPs) via cathodic electrodeposition, while the silver wire was coated with AgCl to create an Ag/AgCl reference electrode within the tip. This miniaturized tool was designed to connect to a mechanical micropipette for microsampling (20–40 μL) from biological and forensic samples, enabling rapid nitrite ion analysis. Compared to standard laboratory techniques such as spectrophotometry and chromatography, this low-cost, portable electrochemical device demonstrated competitive performance in nitrite sensing.

Beyond (micro)­electrode integration, additional strategies and techniques have been employed to enhance the sensitivity and selectivity of pipet-based sensors. These include modifying electrodes with nanomaterials such as gold nanoparticles^38,39^ and coupling analysis with biomolecules such as enzymes.^40,41^ Recently, new materials and fabrication techniques have been introduced to improve pipet-based electrochemical systems. In addition to conductive pastes and wires, conductive polymers and advanced technologies like 3D printing^6,25,42^ have been employed to develop next-generation three-electrode systems within pipet tips. de Oliveira et al.^43^ developed a portable and versatile electrochemical cell containing three integrated electrodes within a small disposable micropipette tip (polyethylene, 10–200 μL volume range). This system was designed to work with an automatic pipet for small-volume stationary voltammetric analysis and with a syringe tip for larger sample volumes in hydrodynamic and static voltammetric analyses. A notable innovation in their work was the use of a 3D printing pen to fabricate the three-electrode system. Using microgrinding, they created three small holes in the pipet tip, into which a conductive carbon black-polylactic acid (CB-PLA) filament (1.75 mm in diameter) was melted to form the electrodes (Figure 2C). External electrical connections were established using flexible conductive copper wires, which were then insulated. The fabricated device was successfully applied for catechol detection in both stationary and hydrodynamic modes, expanding the potential applications of pipet-based electrochemical sensors.

As mentioned earlier, another key advantage of micropipette tip-based electrochemical systems is their small size, lightweight design, and portability.^35^ These characteristics make them highly suitable for on-site sensing and integration with other portable systems, such as potentiostats, smartphones, and portable photometers, which will be discussed in the next section.

A notable example of such integration was demonstrated by Barragan and Kubota,^44^ who designed a handmade mini-potentiostat and coupled it to an electronic micropipette, enabling control and data acquisition via a smartphone. The integrated micropipette–potentiostat–smartphone system is illustrated in Figure 2D. In this study, gold (Au), platinum (Pt), and silver (Ag) wires were used as electrodes. The mini-potentiostat and its software were custom-designed, programmed, and optimized based on previously reported technologies. This smartphone-controlled electrochemical micropipette offers a highly effective solution for on-site, sensitive electroanalysis. This study, along with the demonstrated potential for integrating pipet-based sensors with other portable devices and Internet-connected instruments, suggests promising future directions for research in this field. It also highlights the broad applicability of micropipette-based electroanalytical tools across various domains, including clinical diagnostics, environmental monitoring, food safety, and industrial processes.

Other reported examples show that tip-based miniaturized electrochemical devises can be tailored via modification with nanomaterials, polymers, or biomolecules, enhancing sensitivity and selectivity for diverse analytical targets. Depending on the application, modified tips can also serve either as electrode holders or as fully integrated electrochemical cells for detecting analytes such as drugs, metabolites, or biomarkers. In the first configuration, González-López et al.^45^ developed a 100 μL polypropylene tip housing stainless-steel pins serving as the working, reference, and counter electrodes, with the working electrode coated with carbon ink for in-tip determination of anionic surfactants via methylene blue interaction. The main advantage of this system was its simplicity and straightforward construction, which allowed the pipet tip to act as both the sample container and the electrochemical cell, minimizing handling steps while enabling rapid and reliable detection of anionic surfactants in small sample volumes. Similarly, Guo et al.^46^ integrated a threonine-modified pencil graphite working electrode, Ag/AgCl reference, and platinum counter electrode into a 200 μL pipet tip. This configuration not only reduced sample consumption drastically from 500 to 10 μL but also possessed an adjustable active surface area, which could be tuned by changing the length of the PT/PGE immersed into the cell suspension from 3 to 15 mm. Such tunability provided flexibility for cell-based studies, making it particularly suitable for the analysis of MCF-7 cancer cells and for evaluating the sensitivity of anticancer drug treatments, thus highlighting its relevance in biomedical and pharmaceutical applications. In another holder-based approach, Stanzione et al.^47^ functionalized 200 μL pipet tips with a laccase–hydrophobin fusion protein to create in-tip enzymatic reactors for the rapid oxidation of caffeic acid. The amphiphilic nature of the hydrophobin and its ability to self-assemble at hydrophobic–hydrophilic interfaces were exploited to allow efficient and stable immobilization of laccase. This innovative immobilization strategy provided enhanced enzyme stability and reusability, while maintaining high catalytic activity, thereby improving the robustness of the electroanalytical system and demonstrating the versatility of functional biomolecule integration within pipet tips.

Oliveira et al.^48^ fabricated a robust and low-cost system using 200 and 10 μL pipet tips with platinum wire electrodes modified with functionalized multiwalled carbon nanotubes for analysis in aqueous and ethanol/water mixtures. Despite its simplicity and minimal cost, the proposed device demonstrated analytical performance comparable to more sophisticated electrodes. Moreover, its compatibility with both flowing conditions and organic solvents suggested potential application as an amperometric detector in liquid chromatography or miniaturized milifluidic devices, highlighting its adaptability and promising role in portable and scalable analytical platforms.

In contrast, integrated-cell configurations were demonstrated by Ramasami Sundhar Baabu et al.,^49^ who increased the sensitivity of their device by sputtering silver onto a bare micropipette tip using a radio frequency sputtering technique to obtain electrical contacts on the tip, followed by the hydrothermal growth of ZnO nanostructures, which acted as the active electrode material. This strategy produced a highly sensitive, miniaturized electrochemical sensor for glucose detection. The combination of Ag sputtering and ZnO nanostructure growth offered significant advantages in terms of signal amplification and improved electrode stability, demonstrating the potential of integrated nanomaterial-modified tips for reliable biosensing applications.

Finally, da Silva et al.^50^ developed a three-electrode-integrated 200 μL tip with carbon nanotubes or carbon beads coated with a molecularly imprinted polymer (MIP), compatible with flow injection analysis. This design allowed analysis in low-volume solutions (ca. 10 μL), which is especially advantageous for waste minimization and the development of clean analytical methods in line with green chemistry principles. Additionally, the integration of MIPs imparted high selectivity for the target analytes, demonstrating the potential of such tip-based devices for sustainable, selective, and miniaturized electroanalytical applications.

All these examples of various configurations and modified micropipette tips, including electrode materials, configurations, and sample volumes, are summarized in detail in Table 1, demonstrating the flexibility, adaptability, and potential of these platforms for miniaturized, low-cost, and high-performance electrochemical sensing.

**Table 1:** Summary of Reported Pipette Tip-Based Electrodes Used for Electrochemical Sensing

**target(s)**	**lab-on-a-tip structure**	**linear range(s)**	**limit of detection**	**sample(s)**	**ref.**
nitrite	**body:** pipette tip (100 μL)	20–150, 150–1200 μM	18.40 μM	urine, river water, and pollutant hand (forensic investigations)	^37^
	**working:** Au wire (diameter, 100 μm; length, 4 mm)				
	**reference:** silver wire (diameter, 0.025 mm; length, 4 mm), used as a substrate for fabricating the Ag/AgCl				
	**counter:** -				
	**method:** cyclic voltammetry (CV)				
ascorbic acid	**body:** pipette tip (10 μL)	30–1400 μM	16 μM	cactus body, lemon, lemon juice	^34^
	**working:** carbon fiber (diameter, 10 μm)				
	**reference:** silver alloy wires (diameter, 0.1 mm)				
	**counter:** -				
	**method:** cyclic voltammetry (CV)				
copper ions	**body:** pipette tip (1000 μL) with cotton filter inside	20–300 μg/L	6.3 μg/L	river water	^36^
	**working:** gold wires (125 mm diameter)				
	**reference:** gold wires (125 mm diameter)				
	**counter:** stainless-steel wire (300 mm diameter)				
	**method:** linear sweep- anodic stripping voltammetry				
glucose	**body:** pipette tip (1000 μL) with cotton filter inside	0.1–10 mM	0.17 mM	Coca-Cola and Coca-Cola Zero	^35^
	**working:** gold wire (diameter of 0.03 mm)				
	**reference:** silver wire (diameter of 0.25 mm)				
	**counter:** stainless-steel wire (diameter of 0.3 mm)				
	**method:** chronoamperometry				
lead ion and catechol	**body:** pipette tip (10 μL) electrodes were printed using 3D-pen-printed electrodes.	lead ion: 20–180 μM	lead ion: 0.049 μM	lead ion and catechol: tap water and Artesian water	^43^
	**working:** carbon black-polylactic acid				
	**reference:** carbon black-polylactic acid				
	**counter:** carbon black-polylactic acid	catechol: 5–300 μM	catechol: 0.11 μM		
	**method:** lead ion: square wave – anodic stripping voltammetry				
	catechol: square wave voltammetry				
sodium dodecyl sulfate (anionic surfactant) by its interaction with methylene blue (MB)	**body:** pipette tip (100 μL)	0–30 μg mL^– 1^	1.2 μg mL^– 1^	two different tap waters	^45^
	**working:** stainless-steel pin coated with carbon ink				
	**reference:** stainless-steel pin				
	**counter:** stainless-steel pin				
	**method:** cyclic or linear sweep voltammetry				
MCF-7 cells	**body:** pipette tip (200 μL)	3000–7,000,000 cells mL^–1^			^46^
	**working:** pencil graphite modified by threonine (PT/PGE)				
	**reference:** Ag/AgCl (Sat’d)				
	**counter:** platinum wire				
	**method:** linear sweep voltammetry				
caffeic acid	**body:** pipette tip (200 μL) modified by hydrophobin-laccase chimera Vmh2-PoxA1b commercial screen-printed electrode	0.5–500 μM	1.4 μM	black and green tea with high content of phenolic compounds	^47^
	**working:** carbon				
	**reference:** Ag/Agcl				
	**counter:** Ag				
	**method:** chronoamperometry				
carbendazim	**body:** 2 pipette tips (200 μL) and (10 μL)	250–2500 μM	49 μM	mineral water and two different orange juices	^48^
	**working:** platinum wire (0.5 mm diameter) modified using acidic functionalized multiwalled carbon nanotubes (F-MWCNTs)				
	**reference:** Ag/AgCl/KCl (sat) in pipette tip (10 μL)				
	**counter:** platinum plate (0.3 mm thickness)				
	**method:** differential pulse voltammetry				
chlortoluron	**body:** capillary (0.5 mm internal diameter), pipet tip (100 μL) and conical glass tube	**river water:** conventional (CPE): 2–200 μM miniaturized (m-CPE): 2–200 μM	**river water:** (CPE): 2.8 μM (m-CPE): 0.34 μM	river water and soil	^31^
	**working:** carbon paste				
	**reference:** Ag/AgCl (3 M KCl)				
	**counter:** platinum wire	**soil:** conventional (CPE): 4–100 μM and miniaturized (m-CPE): 2–100 μM	**soil:** (CPE): 3.1 μg/g and (m-CPE): 4.3 μg/g		
	**method:** differential pulse voltammetry and cyclic voltammetry				
	**sample volume: -**				
glucose	**body:** pipette tip (10 μL)	10–200 μM	67.5 μM	-	^49^
	**working:** ZnO at Ag modified pipet tip pipette tip (1000 μL) used as support for working (Ag sputtering)				
	**reference:** Ag/AgCl electrode				
	**counter:** platinum wire				
	**method:** cyclic voltammetry and amperometry				
glucose	**body:** pipette tip (100 μL)	200–800 μM	20 μM	Human blood samples	^33^
	**working:** carbon fiber (diameter: 7 μm) in tip				
	**reference:** Ag/AgCl electrode				
	**counter:** platinum electrode				
	**method:** cyclic voltammetry and amperometry				
	**sample volume:** 20 μL				
hydrogen peroxide	**body:** pipette tip (1000 μL)	0.91–3.33 mM		Buffer solution	^44^
	**working:** Pt, Au, Cu, Ag, and C (cylindrical 0.20 mm^2^)				
	**reference:** Ag wire with Ag/AgCl anodic deposition (cylindrical 4.9 mm^2^)				
	**counter:** platinum wire				
	**method:** cyclic voltammetry				
hydrogen peroxide	**body:** pipette tip (100 μL)	10–150 μM	80 nM		^50^
	**working:** gold or graphite composite				
	**reference:** silver composite				
	**counter:** graphite composite				
	**method:** cyclic voltammetry and amperometry				
tetrahydrocannabinol	**body:** pipette tip (200 μL)		0.18 ± 0.02 ng/mL		^32^
	**working:** CNT or carbon beads coated with MIP (MAA-Co-EGDMA)				
	**reference:** fabricated Ag/AgCl electrode				
	**counter:** platinum				
	**method:** differential pulse voltammetry				

### Optical Detection with Lab-on-a-Tip

Pipette tip-based optical sensing devices are innovative miniaturized platforms that integrate materials such as filter paper, hydrogels, and 3D-printed components for low-cost, rapid detection of a wide range of analytes. These systems utilize colorimetric, fluorescent, and bioluminescent readouts to enable visually interpretable results without complex instrumentation.^51^ By leveraging the structure of standard pipet tips, they offer a compact, user-friendly solution for decentralized diagnostics.

A simple device design employed 200 μL pipet tips (PTs) modified to function as self-contained tools for extraction and visual detection of synthetic food colorants (FCs) in beverages^52^ (Figure 3A). The PTs underwent physical coating followed by layer-by-layer branching reactions, in which repeated condensation reactions built a highly branched polymer network on the inner walls. This process dramatically increased the inner surface area and the number of active binding groups, enhancing the tip’s adsorption capacity by over 300 times after 8–10 layers. As a result, the PTs enabled rapid, in situ solid-phase extraction and direct colorimetric detection of FCs like Allura Red and Brilliant Blue, with high recovery and visual readout eliminating the need for costly instruments.

**Figure 3:**
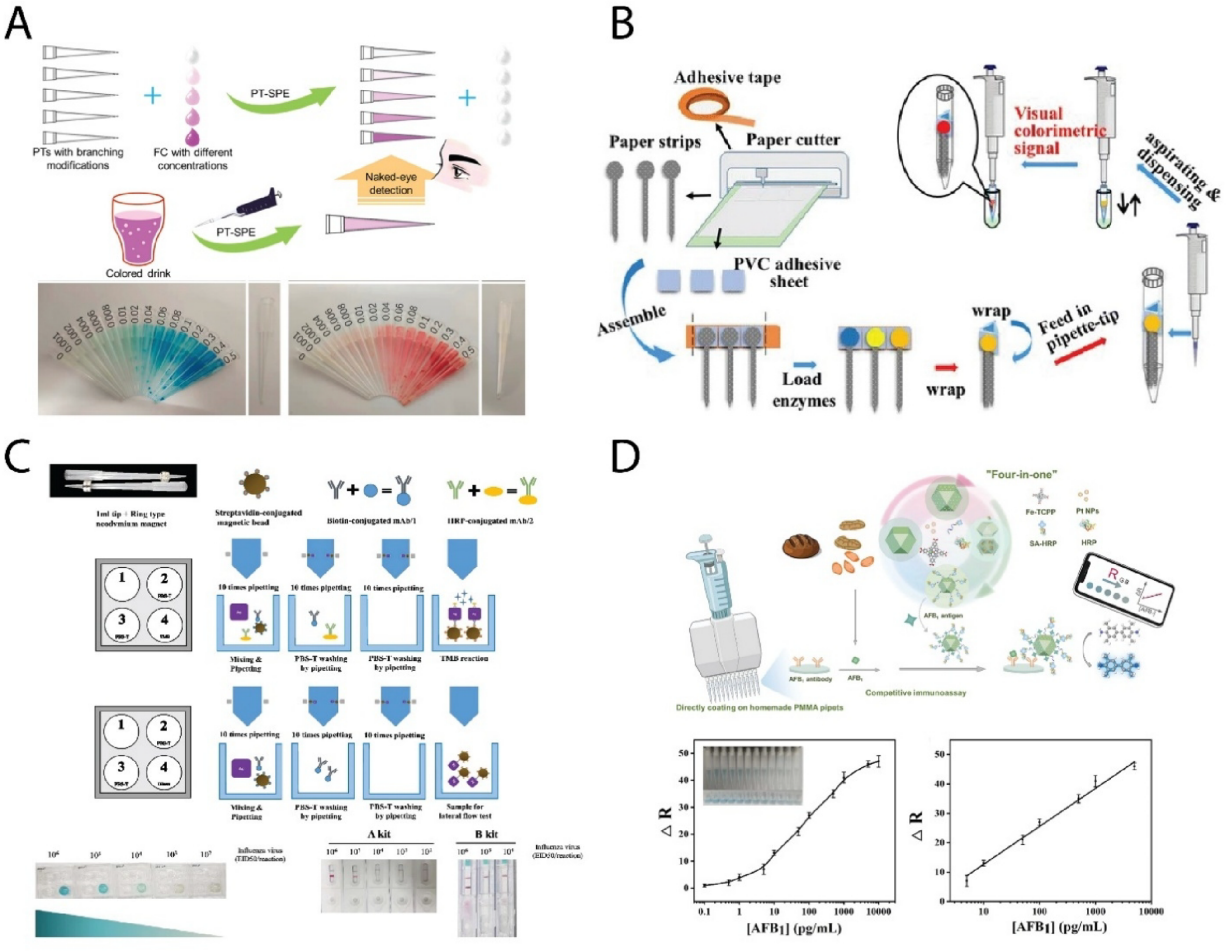
(A) Schematic and visual demonstration of a highly branched pipet tip (PT-SPE) device for semiquantitative naked-eye detection of synthetic food colorants using tip-integrated solid-phase extraction. Color intensity corresponds to analyte concentration after simple pipetting operations. Reproduced from Wang et al. (ref 52). Copyright 2022 published by Elsevier. (B) Assembly and operation of a paper–pipet-integrated device enabling “sample-in, answer-out” visual detection of multiple analytes (protein, glucose, pH) by embedding enzyme-loaded paper strips within a standard pipet tip. Reproduced from Li et al. 2017 (ref 53). Copyright 2017 published by Springer. (C) Magnet-assisted pipetting-based immunoassay platform for rapid detection of influenza A virus, using sequential pipetting of antibody-functionalized magnetic beads, wash steps, and enzymatic colorimetric reaction, performed entirely within a disposable pipet tip. Reproduced from Noh et al., 2019 (ref 54). Copyright 2019 published by Nature Publishing Group. (D) Portable pipet–smartphone detection system for aflatoxin B_1_ in food, based on a ″four-in-one″ nanozyme composite enabling competitive immunoassay and catalytically enhanced colorimetric signal readout directly inside PMMA pipet tips. Reproduced from Hong et al. 2023 (ref 56). Copyright 2023 published by Elsevier.

Another simple yet effective design was suggested by Li et al., who created so-called strips-in-tip configuration by inserting multiple disposable strips of chromatography paper into a standard 1 mL micropipette tip for the detection of multiple analytes.^53^ The strips featured a 20 mm flow path and a 4.5 mm-diameter circular detection zone. By exploiting the porosity of the chromatography paper, the strips also served as storage platforms for reagents such as chromogenic substrates and pH indicators to facilitate analyte detection (Figure 3B). This enables simultaneous colorimetric detection of up to three analytes (glucose, protein, and pH) in urine from a single-step sampling. Visual detection of color generation on the embedded paper strips by the naked eye was possible, thus enabling a multiplexed and contamination-free assay in a semiclosed system. The colorimetric signal enabled both a qualitative analysis, by naked eye, and a semiquantitative analysis, through image processing, by correlating the color intensity to the target concentration or pH changes. The simplicity of the design along with visual readability and the lack of an external power source instrumentation made this approach particularly attractive for decentralized diagnostics.

While many research groups focused on immunoassays within the pipet tip chamber, Noh et al.^54^ introduced a different approach using magnetic bead–based immunocapture via a magnetic pipet tip (Figure 3C). A 1 mL low-binding tip was equipped with external magnetic rings (10 mm diameter, 3 mm thickness, with 4.2 and 6.5 mm central holes) to securely fit the tip and retain magnetic beads. After incubation with HRP-conjugated antibodies and magnetic beads, immune complexes were magnetically held inside the tip during washing and color development. This simplified the workflow and minimized manual handling. The method was applied to detect influenza A nucleoprotein (NP), achieving 100-fold greater sensitivity when combined with lateral flow strips. NP detection was possible at 4.7 ng/μL using only 100 μL of sample. The total assay time was under 20 min, and no specialized instruments were required, making the system ideal for rapid, sensitive diagnostics in point-of-care or field settings.

A notable innovation in optical lab-on-a-tip systems is presented by Kagawa et al.^55^ who developed a compact (40 × 35 × 60 mm), low-cost fluorescence detector (PT-reader) optimized for a standard 20 μL truncated cone-shaped commercial pipet tip. This tip plays a dual role as a disposable reaction vessel and an optical detection chamber, streamlining the PT-based enzyme-linked immunosorbent assay (PT-ELISA). In this assay, anti-IgA antibodies are physically adsorbed onto the tip’s inner wall, enabling in-tip immunoreaction. Fluorescence is generated through HRP-catalyzed conversion of Amplex Red to resorufin. The device achieved a 1.0 ng/mL for IgA in saliva, comparable to standard ELISA but with 10× less reagent. This work highlights a significant advancement in portable diagnostics by combining high sensitivity, minimal sample volume (∼30–40 μL total), and compatibility with unmodified commercial pipet tips, making it particularly suited for decentralized or at-home testing applications.

In another study, other important molecules such as toxins were being detected. Specifically, a simplified and portable biosensor (PSB) was developed for the detection of aflatoxin B1 (AFB1) using a homemade poly­(methyl methacrylate) (PMMA) pipet and a smartphone^56^ (Figure 3D). The PMMA tip played a vital role by providing high protein-binding affinity, allowing direct antibody coating without complex modifications, thereby simplifying the assay and enhancing portability. A multifunctional sea-urchin-like composite catalyst (FSCC) based on Fe–Zr-MOF was used to amplify the signal. This platform enabled highly sensitive colorimetric detection, with a limit of detection down to the picogram per milliliter (pg/mL) level, making it ideal for rapid, on-site toxin screening.

Toward the integration of additive manufacturing for the analysis of more complex samples and biomarkers, Sharafeldin et al.^57^ designed a 3D-printed pipet tip to perform an enzyme-linked immunosorbent assay (ELISA) within the inner walls of the tip itself (Figure 4A). This device enables a single assay but also a multiplex assay with up to eight chambers (tips). The dimensions of the tip were 5.2 cm height and an upper tip diameter of 5.2 mm to fit in pipettes from 50 to 200 μL volume. Through the coating of the inner surface with chitosan hydrogel, the capture antibodies were immobilized and could be used to form a sandwich complex with the analyte and a horseradish peroxidase (HRP)-conjugated second antibody. A colorimetric signal was generated using the tetramethylbenzidine (TMB) chromogenic substrate, and the results were recorded using a smartphone or a CCD camera. The entire system achieved ELISA-like sensitivity in a reaction volume of ∼50 μL and reduced assay time to below 30 min, greatly improving accessibility and potability compared to traditional well-plate ELISA.

**Figure 4:**
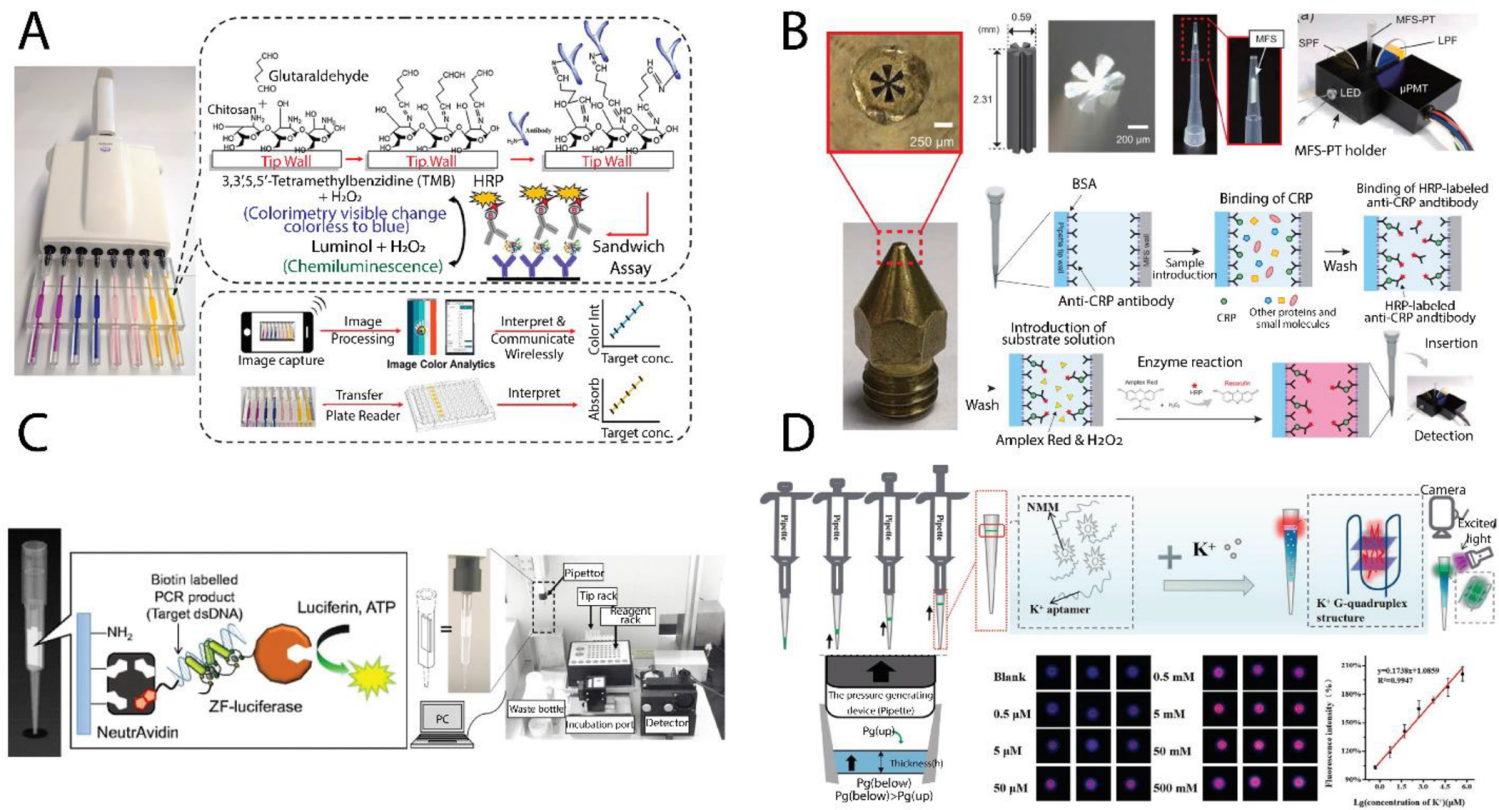
(A) 3D-printed pipet tip–based ELISA platform for telemedicine applications, integrating sample handling, reagent storage, and optical readout within a modular, low-cost tip design compatible with mobile imaging devices. Reproduced from Sharafeldin et al., 2019 (ref 57). Copyright 2019 American Chemical Society. (B) Disposable microfiber-structured pipet tip designed for rapid on-site ELISA applications; antibodies are immobilized on microfiber bundles inside the tip, enabling enhanced surface area for binding and fluorescence-based detection. Reproduced from Nakamura et al., 2025 (ref 58). Copyright 2025 Elsevier. (C) Pipette tip biosensor incorporating a zinc finger–luciferase fusion protein for the bioluminescent detection of double-stranded DNA sequences, with all binding and washing steps automated inside the tip and readout performed by a photomultiplier. Reproduced from Takano et al., 2017 (ref 60). Copyright 2017 Springer. (D) Smart pipet tip–based microhydrogel membrane sensor capable of specific fluorescent detection of small molecules, designed for ease of integration with tip-based liquid handling workflows. Reproduced from Li et al., 2022 (ref 64). Copyright 2022 Elsevier.

Complementary to field of additive manufacturing, to enhance binding efficiency, reduce sample volume, and shorten reaction time, Nakamura et al.^58^ developed a microfiber-type structure (MFS) integrated into a 10 μL pipet tip (Figure 4B). The 3D-printed ABS scaffold (2.3 mm × 0.6 mm) increased the surface area for antibody immobilization, enabling a sandwich-type ELISA for detecting C-reactive protein (CRP) in human serum. Using an HRP-labeled detection antibody and fluorescence readout, the system demonstrated improved antigen–antibody kinetics. Compared to conventional ELISA, assay time was reduced to one-third (55 min), and sample volume decreased ∼33-fold. Fluorescence signals were captured in a dark box using an LED excitation source, with emission converted to photocurrent and then to analog signal. This design shows how microstructural enhancements can significantly boost analytical performance in compact, pipet-based biosensing platforms.

In addition to protein quantification, blood urea nitrogen, a widely recognized biomarker for acute kidney injury, has been effectively measured using a pipet tip–based colorimetric biosensor.^59^ In this method, a sample solution containing phenol red is drawn into a pipet tip whose inner wall is coated with immobilized urease. The enzymatic hydrolysis of urea causes a pH shift, leading to a visible color change in the solution. This change can be captured with a smartphone camera and quantified by analyzing the color intensity. The system achieved a detection limit of 1.3 mg/dL, demonstrating sufficient sensitivity for clinical measurement of urea in human serum. Compared to traditional microwell plate assays, this pipet-based approach reduced both sample volume and analysis time by up to 10-fold. With minimal equipment, as only a micropipette and a smartphone are required, it offers a simple, accessible alternative to laboratory-based assays. This makes the method highly suitable for point-of-care diagnostics and field applications, particularly in settings with limited resources or infrastructure.

For the detection of nucleic acids, an all-in-one, pipet-based device has been successfully developed for DNA extraction, purification, and amplification within a single disposable unit.^60^ This fully integrated diagnostic platform utilizes a plastic Pasteur pipet containing a 3 mm filter paper disc for point-of-care detection of *Bacillus cereus* DNA. The pipet’s 0.1 mL tip (2 mm × 10 mm i.d.) and neck (3.5 mm × 25 mm) formed a compact platform for sample handling. The porous, biocompatible filter enabled solid-phase nucleic acid isolation directly from crude samples. After simple pipetting-based lysis and washing steps, isothermal amplification was performed by sealing the tip and placing the device in a heated water bath. The reaction mixture included a colorimetric indicator, allowing visual detection. The pipet-integrated format minimized user steps, equipment needs, and assay complexity, enabling true point-of-care diagnostics.

As an example of bioluminescent detection within lab-on-a-tip systems, Takano et al. developed a pipet tip-based biosensor for the sequence-specific detection of double-stranded DNA (dsDNA) using a zinc finger–luciferase fusion protein^60^ (Figure 4C). The device, referred to as the “Biologi tip” biosensor using a standard pipet tip containing a 4.3 × 9.8 mm APTES-modified glass plate affixed to the inner wall. This tip serves as the detection zone where biotinylated dsDNA targets are captured via NeutrAvidin–biotin binding. The tip format is essential for housing the immobilized DNA and enabling confined detection reactions. The zinc finger–luciferase fusion protein binds the DNA and, upon addition of the PicaGene substrate, emits bioluminescent light detected via a photomultiplier. Nonetheless, the system demonstrates the feasibility of integrating sequence-specific molecular recognition, surface-based immobilization, and bioluminescent signal generation into a pipet tip format, supporting its potential for future applications in nucleic acid diagnostics.

An innovative Au-on-Au tip sensor was developed for the detection of *Salmonella typhimurium* (*Salmonella*).^61^ Initially, a pipet tip was functionalized with a thin gold layer gold and a DNA probe, and then nanoparticles (AuNPs) were immobilized onto using a synthetic nucleic acid probe (NAP). In the presence of target DNA from *Salmonella*, RNase H2 cleaves the NAP, releasing the DNA-conjugated AuNPs. These released AuNPs, which are proportional to the target DNA concentration, are transferred from the tip onto a nylon membrane integrated with an absorbance pad, where they are detected via a smartphone camera. This portable, cost-effective sensor requires no electronic, electrochemical, or optical equipment and provides a detection limit as low as 3.2 × 10^3^ CFU/mL, making it ideal for simple, on-site diagnostics.

Beyond immunoassays and enzymatic-based assays, colorimetric detection within pipet tips has also been applied to the analysis of ionic species in whole blood.^62^ One notable example is a pipet-integrated system featuring a preloaded organic sensing phase composed of ionophores, chromoionophores, and cation exchangers. In this system, a 30 μL pipet tip was filled with a plasticized oil phase containing the ion-selective components. Detection was based on a pH-sensitive chromoionophore, enabling a colorimetric response that could be captured using a smartphone camera. This setup allows for the direct extraction and quantification of clinically relevant ions, such as calcium and potassium, from whole blood samples without the need for plasma separation. The preloaded oil phase within the tip ensures that the measurement is unaffected by the optical properties of whole blood. The system demonstrated high reproducibility and did not require calibration, making it especially well-suited for at-home monitoring of electrolyte levels in patients with chronic conditions.

Another study for selective calcium ion detection within a pipet tip utilized a 300 μL tip containing multilayered agarose hydrogels embedded with calcium-selective organosilica nanoparticles.^63^ Three gel layers were formed sequentially with volumes of 2, 3, and 5 μL (top to bottom) before cooling. The chromoionophore within the nanoparticles exhibited a visible color change, from blue to red, in response to varying calcium ion concentrations (0.1–0.5 mM). The agarose gel acted both as a reagent reservoir and as a filter to exclude particulates like red blood cells. Detection was distance-based: 5 μL of sample was added, and color progression was imaged using a digital camera and quantified via ImageJ, offering a simple, portable, and cost-effective sensing method.

In a complementary gel-based approach for ion detection, Li et al. developed a potassium ion-sensitive gel inside a 10 μL pipet tip by aspirating a defined volume of gel precursor^64^ (Figure 4D). The gel consisted of a G-rich DNA probe and a fluorescent dye. After intake, a small air gap was drawn in to position the solution, and the tip was left to cool naturally at room temperature, forming a stable hydrogel. In the presence of potassium ions, the DNA folds into a G-quadruplex structure that binds the fluorescent dye, resulting in enhanced fluorescence. The fluorescent images were captured via a smartphone for quantitative image analysis. The pipet tip here served as the detection zone integrated with the gel, eliminating the need for expensive equipment and complex assay designs. This work exemplifies how smart materials and molecular recognition elements can be seamlessly integrated into pipet-tip platforms, extending their capabilities beyond colorimetry into the realm of soft-matter fluorescence sensing.

Collectively, as shown in Table 2, these diverse strategies highlight the remarkable adaptability of pipet tip-based platforms for optical sensing. By integrating smart materials such as filter paper, hydrogels, and 3D-printed scaffolds, along with innovative designs like magnetic immune-capture and embedded sensing phases, these devices enable sensitive, low-cost, and often multiplexed detection in compact, portable formats. Exploiting the inherent convenience and structure of standard pipet tips, lab-on-a-tip systems are emerging as powerful tools for next-generation point-of-care diagnostics, environmental monitoring, and food safety analysis.

**Table 2:** Summary of Reported Pipette Tip-Based Devices for Optical Sensing^a^

**target(s)**	**lab-on-a-tip structure**	**linear range**	**limit of detection**	**sample(s)**	**ref.**
glucose, protein, pH	**body:** pipette tip (1 mL) with inserted paper strips consisting of a 20 mm flow path for sampling and a 4.5 mm-diameter circular detection zone	qualitative (multiplexed color detection)	not specified	artificial urine	^53^
	**method:** colorimetric via chromogenic enzymatic assays and pH indicators	semiquantitative glucose: 0–7 mM			
	**readout:** naked eye/smartphone	BSA: 0–50 mg/mL pH: 5–9			
	**sample volume:** 100 μL				
cancer biomarkers (IGF-1; PSA; VEGF; CD-14)	**body:** single assay: 3D-printed pipet tip (50–200 μL) of 5.2 cm height and 5.2 mm upper diameter. Multiplex assay: multichamber (8-tips). Internal tip surface coated with chitosan for antibody immobilization **Method:** colorimetric/chemiluminescent ELISA. **Readout:** smartphone, CCD, or plate reader. **Sample volume:** 50 μL	N/A	plate reader: PSA 5 pg/mL, VEGF 25 pg/mL, IGF-1 4 pg/mL, CD-14 4 pg/mL. CCD camera: PSA 1 pg/mL. Smartphone: IGF-1 20 pg/mL, VEGF 50 pg/mL	spiked human serum	^57^
C-reactive protein	**body:** clear pipet tip 10 μL integrated with ABS-based microfiber structure of 2.31 mm height and 0.59 mm diameter. **Method:** fluorescence-based sandwich-type ELISA. **Readout:** fluorescence translated to photocurrent. **Sample volume:** 3 μL	0–10 ng/mL	0.78 ng/mL	buffer	^58^
influenza A nucleoprotein	**body:** 1 mL pipet tip integrated with neodymium magnets measuring 10 mm in diameter and 3 mm in thickness, featuring central holes of 4.2 and 6.5 mm. **Method:** colorimetric. **Readout:** naked eye/image analysis. **Sample volume:** 100 μL	0.047–47 ng/μL	4.7 ng/μL	various species biofluids	^54^
DNA of *Bacillus cereus* pathogen	**body:** 0.1 mL disposable Pasteur tip with dimensions of 2 mm × 10 mm inner diameter and the neck size of 3.5 mm × 25 mm integrated with a 3 mm filter paper disc. **Method:** colorimetric using an indicator in the amplification reaction mixture. **Readout:** naked-eye, real-time fluorescence system for quantification. **Sample volume:** 100 μL	10^–13^–10^–10^ M	10^–13^ M genomic DNA	milk	^65^
Synthetic food colorants	**body:** 200 μL pipet tip modified by physical coating with a highly branched polymer network in inner walls. **Method:** colorimetric **Readout:** naked-eye, smartphone/image analysis. **Sample volume:** 200 μL	0.001–2.0 mg/mL	10^–3^ mg/mL	beverages	^52^
aflatoxin B1	**body:** 1 mL poly(methyl methacrylate) (PMMA) pipet tip with immobilized antibody in the internal walls. **Method:** colorimetric competitive immunoassay. **Readout:** smartphone/image analysis. **Sample volume:** 5 μL (200× dilution)	1–500 ng/mL	1.57 pg/mL	Spiked bread and peanut	^56^
food-borne pathogen Salmonella typhimurium	**body:** 20 μL pipet tip functionalized with a thin gold layer and immobilized specific DNA probes in the internal walls. **Method:** colorimetric AuNP-based hybridization cleavage assay on nylon membrane. **Readout:** smartphone/image analysis. **Sample volume:** 20 μL	2.6 × 10^2^–10^8^ CFU g^–1^	1.4 × 10^3^ CFU g^–1^	round beef and chicken meat	^61^
immunoglobulin A	**body:** small-sized (40 × 35 × 60 mm) fluorescence detector (PT-reader) using a transparent 20 μL commercial circular truncated cone-shaped pipet tip as a reaction/detection vessel. The tip is inserted into a custom holder made of PDMS mixed with 5% carbon black. **Method:** fluorescent ELISA. **Readout:** fluorescence detector. **Sample volume:** ∼40 μL	0–28 ng/mL	1.0 ng/mL	human saliva	^55^
dsDNA derived from *E. coli* O157	**body:** custom pipet tip fitted with a 4.3 × 9.8 mm APTES-modified glass reaction plate for biotin-based immobilization chemistry. **Method:** bioluminescence generated by zinc finger protein–luciferase fusion. **Readout:** bioluminescence intensity measured by a photomultiplier. **Sample volume:** ∼500 μL	1.2 × 10^2^–10^5^ copies	120 copies	buffer	^60^
blood urea nitrogen	**body:** clear pipet tip 10 μL with immobilized capture antibodies in the inside walls. **Method:** colorimetric. **Readout:** smartphone/image analysis. **Sample volume:** 2.5 μL	1.3–25 mg/dL	0.013 mg/mL	serum	^59^
Ca^2+^, K^+^	**body:** 30 μL pipette tip with preloaded plasticized oil containing ionophore mix and ion exchanger. **Method:** colorimetric via a pH-sensitive chromoionophore. **Readout:** smartphone camera. **Sample volume:** 2–10 μL	Ca^2+^: 0.5 μM–2 mM. K^+^: 0.5 μM–500 mM	N/A	whole blood	^62^
K^+^	**body:** 10 μL pipet tip with in situ formed smart hydrogel membrane (130 μm thickness) in the conical channel. **Method:** fluorescence detection using DNA aptamer and dye. **Readout:** smartphone camera or fluorescent detector. **Sample volume:** 2–10 μL	0.5 μM–500 mM	0.5 μM	human serum	^64^
Ca^2+^	**body:** 300 μL pipet tip filled with three layers of agarose hydrogel; each layer contained calcium-selective organosilica nanoparticles; and gels were loaded in sequence from top to bottom with 2, 3, and 5 μL of gel solution, solidified upon cooling. **Method:** colorimetric via chromoionophore. **Readout:** smartphone/image analysis. **Sample volume:** 5 μL	0.1–0.5 mM	N/A	chicken blood, serum	^63^

a N/A: not applied, ELISA: enzyme-linked immunosorbent assay, PSA: prostate specific antigen, VEGF: vascular endothelial growth factor, CD-14: insulin-like growth factor-1 (IGF-1), CD-14: cluster of differentiation-14, ABS: acrylonitrile butadiene styrene, PDMS: polydimethylsiloxane, APTES: 3-Aminopropyl triethoxysilane, AuNPs: gold nanoparticles.

## Conclusions and Outlook

Pipette tip-based analytical devices have established themselves as highly versatile, cost-effective platforms for miniaturized chemical and biological sensing. Current technologies predominantly focus on two main approaches: electrochemical sensors, where pipet tips act as holders or integral components of (micro)­electrodes, and optical sensors that utilize colorimetric, fluorescence, and bioluminescent detection methods. These systems have successfully targeted a wide array of analytes, including proteins, pathogens, DNA, ions, and toxins. The incorporation of functional materials such as filter paper, hydrogels, polymer networks, and magnetic beads has significantly enhanced sensitivity, selectivity, and reagent storage capabilities. Furthermore, advances in nanomaterials and enzyme integration have elevated analytical performance, while smartphone compatibility has facilitated real-time, decentralized diagnostics, making these devices especially suitable for point-of-care and resource-limited environments.

Despite these promising developments, notable gaps remain. Most pipet tip sensors currently operate in a unimodal manner, focusing exclusively on either electrochemical or optical detection. The creation of multimodal lab-on-a-tip systems, which integrate complementary sensing modalities within a single pipet tip, remains an underexplored frontier.^66^ Such integration promises richer, more reliable analytical data, broadening the detectable analyte spectrum and enhancing diagnostic precision.

Another area ripe for advancement is the full exploitation of additive manufacturing technologies, particularly 3D printing, in pipet tip sensor fabrication.^6^ While 3D printing has been primarily employed for device housing or scaffolding, its potential for directly fabricating functional sensing elements, electrode architectures, and integrated microfluidic features inside pipet tips is still largely untapped. Embracing advanced printing techniques and novel printable materials could revolutionize sensor design by enabling highly customizable, complex, and reproducible structures with improved performance and multifunctionality.

In conclusion, future research should prioritize the development of integrated multimodal lab-on-a-tip platforms that synergistically combine electrochemical and optical detection methods. Simultaneously, leveraging the full capabilities of 3D printing and other additive manufacturing techniques, not merely for device housing but for sensor fabrication itself, will be essential to propel the field forward. Addressing these challenges will accelerate the emergence of next-generation pipet tip-based sensors that are more sensitive, versatile, and adaptable, meeting the increasing demand for sustainable, accessible, and high-performance analytical tools across biomedical, environmental, and food safety domains.
